# Maladaptive Daydreaming in Relation to Linguistic Features and Attachment Style

**DOI:** 10.3390/ijerph19010386

**Published:** 2021-12-30

**Authors:** Rachele Mariani, Alessandro Musetti, Cinzia Di Monte, Kerri Danskin, Christian Franceschini, Christopher Christian

**Affiliations:** 1 Department of Dynamic and Clinical Psychology and Health Studies “Sapienza”, University of Rome, Via degli Apuli 1, 00185 Rome, Italy; cinzia.dimonte@uniroma1.it; 2 Department of Humanities, Social Sciences and Cultural Industries, University of Parma, 43125 Parma, Italy; alessandro.musetti@unipr.it; 3 Licensed Psychologist in Private Practice, Princeton, NJ 08544, USA; kerridanskinphd@gmail.com; 4 Department of Medicine and Surgery, University of Parma, 43125 Parma, Italy; christian.franceschini@unipr.it; 5 Department of Psychology, City College, City University of New York, 160 Convent Avenue, New York, NY 10031, USA; cctian0702@gmail.com

**Keywords:** maladaptive daydreamers, attachment, linguistic measures, multiple code theory

## Abstract

(1) Background: Maladaptive daydreaming (MD) is a concept that describes a significant imaginary activity that replaces human engagement and/or interferes with academic, interpersonal, or vocational functioning. We explored the interaction between attachment style, reflective functioning (RF), and the narrative dimension of MD. (2) Methods: 414 adults completed an online survey, including socio-demographic variables, the 16-item Maladaptive Daydreaming Scale, the Symptom Checklist-90-Revised, Relationship Questionnaire, and Reflective Functioning Questionnaire. Participants were asked to write a short description of the most representative episodes of their daydreams. Eighty-four participants were identified as maladaptive daydreamers (MDers). (3) Results: A set of *t*-tests between MDers and non-MDers group showed differences in attachment dimensions, RF, and linguistic measures. A linear regression model with Global Severity Index (GSI) of the revised Symptom Checklist-90 as the dependent variable, and psychological scales as independent variables showed that the MD score was the strongest predictor of GSI. Regarding differences between the two groups in linguistic measures, the MDers showed more use of reflection and sensory-somatic words, and a smaller number of affective words. (4) Conclusions: These results support the idea that the MD is a process connected to psychopathological mechanisms, probably to a sub-symbolic activation, and to dysfunctional self–other relational patterns that are difficult to integrate.

## 1. Introduction

According to a growing body of literature, maladaptive daydreaming (MD) is characterized as “extensive imagination activity that replaces human interaction and/or interferes with academic, interpersonal, or vocational functioning,” [1]. Somer’s seminal work on six maladaptive daydreamers (MDers) [1] featured a description of captivity, rescue and escape, and idealized self as central MD motifs. MDers can lose themselves for hours in vivid, highly structured dreams, frequently with a strong sense of being present in the daydream.

MD is a subset of the greater cognitive phenomena of daydreaming (i.e., a waking fantasy activity), which is a common, healthy mental activity that 96 percent of Americans engage in [2]. According to Killingsworth and Gilbert (2010) [3], this brain process accounts for over half of all human thought, and the average person appears to have hundreds of daydreaming episodes per day [4].

Daydreaming may operate on a continuum from healthy daydreaming to MD, which has been conceptualized as a psychological dysfunction attendant to trauma [1]. Historically, Freud believed that the function of the daydream was the same as that of night dreams—that is, wish-fulfilment. Freud (1908) [5] wrote, “it was no longer difficult to recognize that night-dreams are wish-fulfilments in just the same way as day-dreams—the phantasies which we all know so well” (p. 149).

Most contemporary psychoanalysts would agree that beyond wish fulfilment, adaptive daydreaming can serve a host of purposes, such as to reverse narcissistic injuries, spur creativity and artistic pursuits, or heighten self-esteem and pleasurable affects. When daydreaming is not determined by defensive functions, it can be flexible and contain elements of reversible dissociation and regression. Along these lines, Kris (1952) [6] famously described how certain regressions can be in the service of the ego, in contrast to other regressions which evidence an ego that is overwhelmed.

“We have now to elucidate in greater detail the relations of wit and caricature to dreams: in dreams, the ego abandons its supremacy, and the primary process obtains control, whereas in wit and caricature the process remains in the service of the ego” (p. 177). This phrase alone suffices to indicate that the problem at hand is a more general one. The distinction between an ego overwhelmed by regression and ‘regression in the service of the ego’ covers a large and imposing range of mental experience.

Other authors have noted how daydreams can serve as a form of motoric self-control, self-restraint, and delay of gratification [7,8,9]. In its maladaptive form, daydreaming can be rigid and compulsory, leading to self and object fragmentation and experiences of dissociation marked by distressing self-states [10]. MD may be determined by defensive needs and may represent an escape into fantasy and avoidance. Winnicott believed that excessive daydreaming and fantasizing interferes with action and negatively affects dreaming [11].

### 1.1. Maladaptive Daydreaming and Attachment

Decades of research, beginning with the work of John Bowlby [12,13,14], suggests that the content of fantasies and the ways in which those fantasies impact any individual are shaped in part by that person’s state of mind with regard to attachment. Research in the field of attachment has explored Bowlby’s assertion that the nature of a person’s early experiences of receiving care can impact later interpersonal and behavioral patterns [15,16,17] as well as both mental and physical health [18,19,20,21,22,23]. Specifically, attachment theory posits that the presence or absence of a “secure base” caregiver to whom a young child can return for contact, comfort and a sense of safety, can significantly change the way that young child views themself and others throughout their life [24]. Attachment researchers categorize adult attachment patterns in terms of attachment security versus insecurity (dismissive, avoidant, preoccupied, fearful, unresolved, etc.).

Any connection between attachment and MD is as of yet largely unexplored in the empirical literature, but given the nature of MD—with its vivid, imagined interpersonal elements and its dissociative quality—theoretical connections are easy to make. A recent study [25] aimed at exploring attachment characteristics among MDers and normal daydreamers showed a specific attachment style among MDers, characterized by ambivalent-fearful attachment characteristics, while normal daydreamers showed secure-independent attachment styles. In that study, attachment style was assessed by the Attachment Style Questionnaire, which showed MDers appear to have ambivalent feelings about their relationships, perceive themselves as less valued than others, and believe that others will love and respect them less. They also have trouble believing that they can rely on others when they need help.

In this paper, we explored the idea that individuals who evidence an insecure attachment pattern would be more likely both to isolate from others (insecure/dismissing) in fantasy worlds and to construct fantasies about interpersonal interactions to address their underlying attachment needs, sometimes in unboundaried ways that undermine their functioning (insecure/preoccupied).

### 1.2. Maladaptive Daydreaming and Reflective Functioning

A person’s capacity to effectively understand and navigate social interactions is dependent in part on the skill of reflective functioning (RF) [26]. Also referred to as mentalization, RF is the skill that allows an individual to relate with others and understand their behavior via awareness of mental states like feelings, desires, wishes, and intentions. When we understand, for example, that a child is being destructive with their toys because they are overwhelmed or tired, we are employing a theory of mind, and engaging in RF. This skill is understood to develop best in a secure caregiving environment; when attachment security is threatened, RF development too is jeopardized [27]. In fact, the findings of some studies established a strong relationship between RF and attachment patterns in a variety of ways [26,27]: people who scored high in RF were also more likely to be characterized as secure/autonomous on the attachment interview. RF ratings were also highly positively correlated with coherence ratings on the adult attachment interview.

Like attachment security, RF is important in a person’s sense of self, as demonstrated by its negative relationship with borderline personality disorder [26,28]. Impairment in RF also may be implicated in other mental health problems, including psychosis, mood and anxiety disorders [29] and addictive behaviors [30].

As with attachment, the link between MD and RF is as yet underexplored in the research literature. In this study, we used Fonagy et al.’s Reflective Functioning Questionnaire (RFQ; 2016) [31] to explore how this dimension of cognitive and interpersonal functioning might relate to MD. Theoretically, RF could be relevant in the understanding of MD because it helps individuals distinguish between what is real and what is not and also to understand what comes from one’s own mind, and what comes from interpersonal interactions [32]. Specifically, we explored here how MD may relate to the factor of uncertainty about mental states as measured by the RFQ, which have been shown to be correlated with empathy, mindfulness and perspective-taking [31].

### 1.3. Maladaptive Daydreaming from Multiple Code Theory Perspective

Multiple Code Theory (MCT) is a general theory of emotional information processing based on recent research in cognitive psychology, psychoanalysis, and affective neuroscience [33]. According to Bucci [34,35], human beings use three basic ways to elaborate information and build up images and representations: (1) the subsymbolic; (2) nonverbal symbolic; and (3) verbal symbolic processing systems. In the subsymbolic system, information is processed simultaneously in a comprehensive and analogical modality, and on a continuous dimension, which organizes the affective core. This system is involved in nonverbal communication such as recognizing a familiar voice, conducting physical activities, and producing creative work. The nonverbal symbolic system uses discrete pictures or representations that emerge from a continuous subsymbolic experience process. Finally, in the verbal symbolic system these images and representations are decoded into words. Because each system keeps its own uniqueness and operates in parallel with the others, any recoding process from one system to another cannot include all material. According to Bucci [34] these three types of processing systems are interconnected by the Referential Process (RP), a series of functional and bidirectional stages representing the process of integrating nonverbal material into a form that can be communicated to others through language [34,36]. RP allows one to communicate emotional experience to others, and to perform a function of self-mutual emotional regulation. When the RP is interrupted (e.g., due to specific internal conflict or trauma) the verbal and non-verbal systems within emotional schemas become disconnected.

We can hypothesize that in MD there is a disconnection between verbal and non-verbal systems, with massive use of the nonverbal symbolic system, and that this disconnection creates difficulties in regulating emotional experience in everyday life.

According to Pennebaker and Chung (2007) [37], the degree to which individuals can cognitively organize an event into a coherent narrative is a marker that the specific experience has achieved a knowledge status. In some cases, through language analysis, it is possible to determine the degree to which a person has come to know their emotions and experiences. The Linguistic Inquiry and Word Count (LIWC) [38] a computerized text analysis program, allows for the analysis of linguistic patterns underlying various psychological constructs. Using a bottom-up, word count-based approach, LIWC operates by comparing each word of a text to an internal dictionary consisting of linguistic and psychological dimensions. LIWC’s ability to detect meaning in a range of experimental scenarios, including showing attentional focus, emotionality, social interactions, thinking styles, and individual differences, has been demonstrated empirically [39].

In sum, MD is a clinical condition consisting of high involvement in daydreaming that has been under-studied but is drawing the attention of researchers. Very few qualitative analysis studies of the content of daydreaming have been published. The first qualitative research [1] investigated the nature and experience of MD while also posing questions about the themes, dynamics, and meanings of MD. As regards the themes, five emerged: violence; idealized self; power, and control; captivity, rescue, and escape; sexual arousal. Another recent study [40] focusing on content analysis of MD aimed to systematically investigate the relationship between a history of childhood trauma among individuals with MD and the content of their daydreams. In that study, various forms of childhood abuse and neglect were linked to morbid imagery and trauma-related re-enactment behaviors, while emotional abuse was linked to dreams about emotional suffering.

The aim of this study is to explore attachment style, RF, and the narrative dimension of maladaptive daydreams. To our knowledge, this is the first study to analyze linguistic features of MD’s content.

We hypothesize that:

(a) MDers will have greater insecure attachment styles, hypomentalization and difficulties in the processes of symbolizing emotional experiences in narratives;

(b) A greater presence of MD symptoms, hypomentalization and insecure attachment styles will predict the presence of greater psychopathological symptoms.

## 2. Materials and Methods

### 2.1. Participants

The sample consisted of 414 (305 female) participants; mean age: 30.36; SD: 12.47. As regards marital status, 132 participants were single, 156 were in a stable relationship, 114 were married, and 15 were divorced. The highest educational level attained by the participants was middle school (19), high school (182), bachelor’s degree (116), master’s degree (84), Ph.D. or specialization (13).

### 2.2. Measures

#### 2.2.1. Socio-Demographic Questionnaire

A socio-demographic questionnaire was designed to collect information concerning age, social status, education level, occupational activity.

#### 2.2.2. Symptom Checklist-90-Revised

The Symptom Checklist-90-Revised (SCL-90-R) [41,42] is a 90-item self-report questionnaire that assesses mental and physical symptoms from the preceding week. Respondents rate their experience of discomfort relative to each item using a 5-point Likert scale format: 0 (not at all), 1 (a little bit), 2 (moderately), 3 (quite a bit), and 4 (extremely). Examples of items from the SCL-90-R include, “Pains in heart or chest” and “Feeling weak in parts of your body.” The Global Severity Index (GSI) score represents overall mental and physical discomfort, while the SCL-90-R consists of nine subscales. The questionnaire showed adequate test-retest reliability, internal consistency, and concurrent and discriminant validity. In the present study, Cronbach’s alpha of the total scale was 0.97.

#### 2.2.3. Maladaptive Daydreaming Scale-16 Items (MDS-16)

The Italian version of MDS-16 [43,44] was used. MDS-16 is a self-report measure made up of 16 items aimed at identifying the presence of maladaptive daydreaming. Respondents are asked to answer the MDS-16 items on a scale ranging from 0 to 10 (0 = never/none of the time; 10 = all the time/extreme amounts). The following are some examples of MDS-16 items: “Some people feel a need to continue a daydream that was interrupted by a real-world event at a later point. When a real-world event has interrupted one of your daydreams, how strong was your need or urge to return to that daydream as soon as possible?”; “When you know you have had something important or challenging to pay attention to or finish, how difficult was it for you to stay on task and complete the goal without daydreaming?” Authors of this questionnaire suggested that a cut-off value of 51 best discriminates between cases and non-cases of self-diagnosed MD. Cronbach’s alpha of the total scale was 0.93.

#### 2.2.4. Relationship Questionnaire (RQ)

The Italian version of the Relationship Questionnaire (RQ) [45,46] was used to measure attachment styles. The RQ is a single item measure made up of four short paragraphs, each describing a prototypical attachment pattern as it applies in close adult peer relationships. Each item corresponds to a specific attachment style: secure, preoccupied, fearful, and dismissing. Participants are asked to rate their degree of correspondence to each prototype on a 7-point scale. The subject is invited to respond from a dimensional and categorical perspective. To begin, each of the four items must be given a 7-point Likert scale rating, and then the indication of self-description among the four items must be evaluated. This questionnaire consists of two subscales that describe the positive or negative models of self and others through the four types of attachment. For the self-model subscale, higher scores will refer to higher anxiety and more negative models of self, whereas for the other-model subscale, higher scores will refer to higher avoidance and more negative models of the other.

#### 2.2.5. Reflective Functioning Questionnaire (RFQ)

The Italian validation of the Reflective Functioning Questionnaire (RFQ) [31,47] was used to assess mentalization. The RFQ comprises two subscales measuring the degrees of uncertainty (RFQ_U) and certainty (RFQ_C) about mental states. The RFQ_U subscale consists of six items such as “Sometimes I do things without really knowing why.” Items are scored by the participant on a 7-point Likert scale (ranging from “completely disagree” to “completely agree”); a high score on this scale reflects hypomentalizing (i.e., a lack of knowledge about mental states), while a low score reflects adaptive acknowledgement of the opaqueness of one’s own mental states, which is indicative of real mentalizing. The RFQ_C subscale consists of six items, such as “I don’t always know why I do what I do” and a low score indicates hypermentalizing; a high score indicates adaptive levels of mental state certainty. In the present study, Cronbach’s alpha of the RFQ_U was 0.72 and Cronbach’s alpha of the RFQ_C was 0.70.

#### 2.2.6. Computerized Referential Process Linguistic Measures

The Discourse Attribute Analysis Program (DAAP) [48,49] is a software program that compares any type of text with lists of words (referred to as dictionaries) in order to either determine the proportion in which those words are present in the text or to determine the level to which several constructs pertaining to the referential process (e.g., referential activity) are associated with a word. The latter is done through the use of “weighted” dictionaries, in which specific words that more strongly relate to the core components of a construct will have greater “weights” than words that are less strongly connected with the construct. DAAP reads texts, compares them word by word to one or more dictionaries, and calculates a weighted average of the dictionary scores for each speaker and each turn of speech, for each text, and for each session. In our study, we used the following dictionaries validated for the Italian language:

The Italian Weighted Referential Activity Dictionary (IWRAD) is a computerized measure of RA [50] in the Italian language. It contains a list of 9596 frequently used Italian words, each assigned a weight between 0 and 1, with 0.5 as the neutral value. A high score represents a high level of RA, which corresponds to a high level of concreteness, specificity, clarity, and imagery in the speech sample. Part of the value of the IWRAD derives from its power to assess linguistic style (rather than only focusing on content) and to represent the unintended aspects of emotional involvement. For a deeper discussion on the method of building a weighted dictionary like IWRAD.

The Italian Mean High-Weighted Referential Activity Dictionary (MH-IWRAD) is a data point that is calculated using IWRAD scores. It is defined as the Referential Activity Intensity Index, essentially a measure of high intensity of emotional engagement emerging from speech [50]. It indicates the extent to which the IWRAD exceeds the mean. It is obtained by looking only at the words with IWRAD scores lying above the mean and then computing, for only those words, the average IWRAD scores. This is perhaps best understood as a measure of upward oscillations in RA scores.

The Italian Reflection Dictionary (IRefD) consists of Italian words referring to both cognitive or logical functions and communication processes that imply the use of cognitive functions [50]. It is a measure of abstract reflection and distancing from emotional experience and corresponds to the proportion of IRefD words present in the speech sample.

The Italian Sensory Somatic Dictionary (ISensD) is a list of Italian words related to the body and bodily activities, and to sensory processes and/or descriptions of symptoms [51]. The number of ISensD words in a speech sample is a measure of the arousal of bodily, sub-symbolic aspects of emotion schemas.

The Italian Sum Affect Dictionary (ISAffD) [51] contains Italian words concerning how people feel and communicate feelings directly. It includes emotion labels, functions associated with affective arousal, and words indicating an emotional response, either positive or negative. ISAffD consists of four sub-dictionaries related to domains of affect: positive affect (IPAffD), negative affect (INAffD), neutral affect without a specific valence (IZAffD), and the sum of the other measures (ISAffD).

#### 2.2.7. Linguistic Inquiry and Word Count

The Linguistic Inquiry and Word Count (LIWC2015. v1.6; Pennebaker Conglomerates, Inc.: Austin, TX, USA, 2015) software [38] is a computerized program aimed at analyzing data related to the language used in writing reports. LIWC program includes the main text analysis module along with a group of built-in dictionaries. LIWC reads written or transcribed verbal texts, then compares each word in the text against a user-defined dictionary. After the processing module has read and accounted for all words in a given text, it calculates the percentage of total words that match each of the dictionary categories. LIWC2015 v1.6 software has been used together with Italian LIWC_2007 Dictionaries. Specific word categories have been chosen for the purpose of the study: Pronouns I and We; I Verbal and We Verbal; Affective Words; Positive Emotions; Optimism; Negative Emotions; Anxiety; Anger; Sadness; Past Time; Present Time; Future Time, and finally a specific list of all punctuation and symbols used in writing specific list (AllPunc and symbols).

### 2.3. Procedure

The present study was conducted between February and June 2021. The work was carried out in accordance with the code of ethics of the World Medical Association (Declaration of Helsinki) for experiments involving humans. Institutional Review Board approval was granted by the Ethics Committee of Department of Dynamic and Clinical Psychology and Health Studies. Questionnaires were made available online through Google Forms. The participants were enrolled using snowball sampling, and they completed an informed consent with a privacy policy before beginning the questionnaires.

Participants were asked to write a short text passage following this instruction: “Describe one of the most representative episodes of your fantasy in which you feel emotionally involved in everyday life, including specific details of its content”. Participants completed the questionnaires after the writing task. This study was conducted only in the Italian population and in the Italian language.

### 2.4. Statistical Analyses

All statistical analyses were performed using the Statistical Package for Social Science version 26 (SPSS version 26, Armonk, NY, USA). Data are reported as means and standard deviations for continuous variables and as percentages for discrete variables.

According to the MDS-16 cut-off score, the sample was divided into two groups: A no-MD group composed of people reporting MDS-16 total scores of less than 50, and an MD group composed of people reporting MDS-16 total scores of less than 50. Student’s *t*- and Chi-square tests were performed in order to evaluate the homogeneity of the two groups respectively for continuous and discrete variables.

The Student’s *t*-test was also performed in order to evaluate differences in RF, attachment style, linguistic measures, referential process, and LIWC indexes between two groups. A *p* value of less than 0.05 was considered significant.

Lastly, a linear regression analysis was performed to investigate possible predictors of psychopathology (GSI), using variables that were significant from the Student’s *t*-test as predictor variables (age, gender, RFQ Certainty scale, RQ self-model, RQ other-model and, MDS-16 total score).

## 3. Results

Among the 414 participants, the mean score on the global severity index (GSI) was 0.90 (SD = 0.61). For the MDS-16, the mean score was 33.78 (SD = 19.76). Using the MDS-16 cut-off of 50, the sample was divided into two groups: 330 participants whose MDS scores were lower than or equal to 50 (No-MD group) and 84 people whose MDS scores were higher than 50 (MD group). The homogeneity between groups in terms of age was evaluated and a significant difference emerged (t(412) = 3.91; *p* < 0.001), discussed below.

Table 1 and Table 2 report the results of Student’s *t*-test in RF, attachment style, linguistic measures, referential process, and LIWIC indexes between the two groups.

Since significant differences in psychological scales emerged between MD and No-MD groups, a linear regression model was performed. A linear regression analysis having GSI as the dependent variable and age, gender, RFQ Certainty scale, RQ self-model and, MDS-16 total score as predictors was run. This model was significant and predicted 36% of GSI (R2 = 0.361; adjusted R2 = 0.352; *p* < 0.001); gender (beta = 0.137, *p* = 0.001), RFQ Certainty scale (beta = −0.178, *p* <0.001), RQ self-model (beta = 0.243, *p* < 0.001), MDS-16 total score (beta = 0.356, *p* < 0.001) emerged as significant predictors.

## 4. Discussion

Our study explored the relationship between MD, attachment style, and RF and the narrative dimension of daydreams.

MD was linked to age, which is a significant factor. Younger people reported a greater presence of daydreams. This finding is in line with the literature [52,53] and with the evidence that young people are increasingly aware of the phenomenon of maladaptive daydreaming. Given that it is often a self-diagnosed phenomenon, it may be that young people are more likely to notice and report, and willing to share, their experiences of it.

Regarding attachment style, our results showed that the MD group members had a greater prevalence of worried and fearful attachment. In fact, the MD group also had significantly higher scores in the self model RQ scale, which is associated with attachment higher anxiety. This suggests that perhaps in some cases, MD could be an attempt to regulate painful feelings of abandonment and rejection. An interesting result emerges by analyzing the difference between MDers and non-MDers on the RF scale. Here the greatest difference is linked to the RQ-Certainty factor; a very low score on this factor reflects hypermentalizing according to the authors of the scale [31]. This could suggest that MDers hypermentalize less, but several studies have criticized the use of this factor as a true index of hypermentalization in the general population. In fact, it appears that the Certainty factor is instead associated with a good mentalization functioning [23,54,55]. Our study appears to confirm this.

In fact, participants in our MD group endorsed more Uncertainty, which may signal a dimension of hypomentalization, though this finding was not significant.

The most original aspect of our study was the analysis of the texts of daydreams. We asked our participants to share one of their most frequent daydreams, trying to be especially descriptive. There are currently no studies to which we can compare to our results, but our analysis highlights some peculiarities. In order to analyze daydreams we used two sets of the most used computerized text analysis measures—Pannebaker’s LIWC and Bucci’s Referential Process Linguistic Measures. The first finding is linked to the frequent use of the first-person point of view, both in terms of pronouns and verb tense. Many studies show how this form of communication is linked to a depressive dimension, so much so that it is referred to as the specific “Language of Depression” [56,57,58,59]. Accordingly, a significantly smaller number of positive affect words were presented in the MD group, further suggesting a connection to depressed mood. There was also an interesting pattern in which MDers often described a contrast between themselves and others using “I” and “they” or I/they verb conjugations as in, for example, “I do good things but they always do something to destroy that.” The participants from the MD group produced daydreaming descriptions with higher word counts, suggesting perhaps that they were more invested in the writing process and so more detailed dreams emerged. Similarly, the MD group used more Abstract/Reflection words, which are associated with intellectualized process as a defense mechanism [60,61,62,63]. Sensory-somatic words, which describe bodily symptoms, were also higher in the MD group. Generally, the sensory-somatic dictionary is associated with higher levels of Mood Disorder or Psychosomatic aspects [51,56]. Consistent with Multiple Code Theory (MCT), we found a significant result related to the Referential Activity intensity index, which is connected to dysregulation processes and dissociation process [64]. In addition, MDers’ dream narratives contained more future perspective as well as probabilistic hypotheses, with use of the future tense and conditional verbal forms.

Finally, in the Google Form it was not possible to use emojis because the sample wrote with a PC and not with a smartphone, but MDers used punctuation and symbols more frequently than non-MDers, often in the form of emoticons with punctuation such as ;-) or :). This finding raises an interesting question about non-verbal symbolic coding; MCT might suggest that the emotional and integrative needs of the MDers may color their information processing and compel them to enhance the texts with non-verbal communication. Here are examples of two daydreams from the MD group:

 *A.*  *Often….especially when I listen to music…. I happen to come out of the ordinary world and think and reflect on what the future could be like… .. after graduation, ;) in work, and in general with regard to social problems and climate change!! Most of the time I find myself imagining myself as an accomplished person who tries to enhance the territory in which he works and where he was born !!!!!!*
 *B.*  *The most recurring types of fantasies in my daily life have a journey as their central theme. In almost all my day-dreaming I am the protagonist ... Everything usually starts from where I am at that moment in reality (for example in class, at home, at the supermarket etc ...) from there a short scene that introduces the purpose of the journey that can be trivial like a friend who needs to disconnect from studying or more demanding like a catastrophic event that makes it preferable to stay away from large inhabited centers!! [...] Here the dream becomes very vivid, my senses light up, I begin to feel a pleasant breeze: it caresses my face, while it gently shakes the branches of the trees and winds through the blades of grass around me. In addition to an obvious sense of serenity, I feel the priceless certainty of having completed everything that was there before the trip and of being right where I should be; I know that what I am doing right now, sitting in the middle of this show, is not meaningless!!!!*

These passages are rich with non-verbal experience, which MCT would suggest reflects the need to self-regulate emotion [65] and try to connect sub-symbolic experiences to verbal experiences.

As regard our second hypothesis, the results of the linear regression showed that MD is the strongest predictor of the Global Severity Index. This finding highlights a strong connection between psychopathological symptoms and an imagination strategy, which could highlight a certain transversality of extensive fantasy activity as a basic condition of psychological malaise.

## 5. Conclusions

From the analysis of the texts, together with the configuration of the attachment patterns and RF, we can deduce that the affective process is disconnected from the symbolic process. This probably pushes MDers to use this imaginative strategy more frequently as an attempt to symbolize dissociated emotional experiences that are difficult for them to integrate into everyday life. These preliminary results support the idea that the phenomenon of maladaptive daydreaming is a process connected to psychopathological mechanisms, probably to sub-symbolic activation and to dysfunctional self–other relational patterns that are difficult to integrate.

There are many limitations of our study, including the unrepresentative sample in terms of gender; although it is in line with previous studies [66]. Moreover, our method of collecting information through a Google form that does not allow any control or clinical verification. Another limitation is the absence of some information, explored in literature and highly correlated to MD, about our sample (for example internet addiction) [50].

Finally, few studies have been conducted on the narratives of MDers so these findings are exploratory of the phenomenon but cannot be generalized. Possible future directions could include exploring in a more detailed way the formulation of daydreams in relation to specific diagnoses.

## Figures and Tables

**Table 1:** Differences in attachment and reflective functioning between No-MD and MD groups.

	No-MD Group *n* = 330		MD Group *n* = 84			
	M	SD	M	SD	*t*-Test	*p*
Secure_RQ	3.33	1.83	2.69	1.62	2.94	**0.003**
Fearful_RQ	2.83	1.71	3.30	1.89	−2.23	**0.026**
Preoccupied_RQ	2.41	1.68	3.27	1.88	−4.06	**<0.001**
Dismissing_RQ	2.73	1.66	2.89	1.70	−0.76	0.444
Self model_RQ	−0.82	3.67	1.00	4.16	−3.95	**<0.001**
Other model_RQ	0.18	3.63	0.23	3.71	−0.94	0.343
RFQ_Certainty	1.07	0.70	0.76	0.54	3.81	**<0.001**
RFQ_Uncertainty	0.79	0.52	0.92	0.60	−1.87	**0.061**

RQ = Relationship Questionnaire; RFQ = Reflective Functioning Questionnaire.

**Table 2:** Differences in linguistic measures of Referential Process and LIWIC indexes between two groups.

	NO-MD Group *n* = 330		MD Group *n* = 84			
	M	SD	M	SD	*t*-Test	*p*
Linguistic measures of RP						
WordCount	17.336	20.385	23.726	44.966	−1.923	**0.055**
Negative Aff	0.008	0.038	0.013	0.050	−1.016	0.310
Positive Aff	0.038	0.114	0.011	0.031	2.146	**0.032**
Sum of Aff	0.031	0.066	0.028	0.058	0.369	0.711
Neutral Aff	0.004	0.017	0.004	0.015	−0.342	0.732
Abstract/Reflection	0.063	0.094	0.092	0.128	−2.323	**0.020**
Sensory-Somatic	0.051	0.100	0.080	0.112	−2.269	**0.023**
Referential Activity	0.495	0.067	0.489	0.022	−0.402	0.687
RA Intensity Index	0.009	0.021	0.005	0.010	−1.989	**0.047**
LIWC						
I	6.127	7.7329	4.3128	6.5016	−2.889	**0.004**
We	0.161	1.0395	0.1187	0.9953	0.336	0.736
Other	0.048	0.4324	0.0649	0.4445	−0.325	0.745
Possibility	1.765	3.6443	3.3936	5.6164	−3.234	**0.001**
Past	0.802	2.9888	0.6148	2.3761	0.532	0.594
Present	8.838	11.3416	8.9042	11.2308	−0.047	0.961
Future	0.156	0.8409	2.2049	5.8358	−6.152	**0.000**
To Be	0.450	1.7192	0.2696	0.9081	0.930	0.352
To Have	0.935	3.8618	0.4519	1.6908	1.119	0.263
I_Verbal	3.868	8.2293	8.2037	16.9666	−3.350	**0.000**
You_Verbal	0.072	0.8396	0.0126	0.1157	0.648	0.517
He_Verbal	0.870	2.9219	0.6738	1.8503	0.584	0.559
We_Verbal	0.052	0.4228	0.0776	0.7114	−0.415	0.678
Other_Verbal	0.017	0.2157	0.0254	0.2324	−0.324	0.745
They_Verbal	0.261	1.1729	1.2381	4.2330	−3.685	**0.000**
AllPunc and symbols	6.147	9.9735	11.379	28.5098	−2.745	**0.006**

RP = Referential Process; LIWC = Linguistic Inquiry and Word Count.

## Data Availability

The data presented in this study are available on request from the corresponding author.
